# Endothelial Protein Changes Indicative of Endometriosis in Unexplained Infertility, an Exploratory Study

**DOI:** 10.3390/ijms26136485

**Published:** 2025-07-05

**Authors:** Heba Malik, Sirine Zamouri, Samir Akkawi, Siddh Mehra, Rana Mouaki, Thozhukat Sathyapalan, Manjula Nandakumar, Alexandra E. Butler, Stephen L. Atkin

**Affiliations:** 1 School of Medicine, Royal College of Surgeons in Ireland-Medical University of Bahrain, Busaiteen P.O. Box 15503, Bahrain; 22200851@rcsi-mub.com (H.M.); 23202195@rcsi-mub.com (S.Z.); 22200768@rcsi-mub.com (S.A.); 23204412@rcsi-mub.com (S.M.); 22202123@rcsi-mub.com (R.M.); 2 Hull York Medical School, University of Hull, Hull HU6 7RX, UK; thozhukat.sathyapalan@hyms.ac.uk; 3 Research Department, Royal College of Surgeons in Ireland-Medical University of Bahrain, Busaiteen P.O. Box 15503, Bahrain; mnandakumar@rcsi.com (M.N.); satkin@rcsi.com (S.L.A.)

**Keywords:** endothelial cell markers, VCAM, VEGF, sICAM-3, angiopoietin-1, sTie-2, unexplained infertility, vitamin D

## Abstract

Previous research has linked both endothelial protein changes and vitamin D with infertility. This study was undertaken to investigate the association of proteins associated with endothelial function and vitamin D status in the luteal phase at day 21 in a group of non-obese women prior to in vitro fertilization (IVF) with either unexplained infertility (UI) or male factor infertility (MFI). Twenty-five non-obese Caucasian women from a UK academic center with MFI (*n* = 14) and UI (*n* = 11) were recruited. Blood was withdrawn at day 21 of the menstrual cycle at the time of mock embryo transfer. Vitamin D parameters were measured by tandem mass spectroscopy. Off-rate Modified Aptamer (SOMA)-scan plasma protein measurement was undertaken for 20 protein markers of endothelial dysfunction. Baseline demographics did not differ between groups and parameters of response following IVF did not differ. Vitamins D_2_ and D_3_, and 1,25 Vitamin D_3_ did not differ between groups. In UI, markers of endothelial activation/dysfunction were investigated; vascular cell adhesion molecule 1 (VCAM-1) decreased and this is associated with endothelial stress; vascular endothelial growth factor (VEGF) decreased and this may suggest impaired endometrial angiogenesis; while intercellular adhesion molecule 1 (ICAM-3) increased (*p* < 0.05) and is associated with increased immunological activity. A marker of vascular integrity, angiopoietin-1, increased while soluble angiopoietin-1 receptor (sTie-2) decreased (*p* < 0.05), suggesting increased vascular development. Endothelial markers of inflammation, coagulation, and endothelial progenitor cells were unchanged. Vitamin D and its metabolites show no relationship to UI, but endothelial activation/dysfunction and vascular integrity changes in VCAM-1, VEGF, sICAM-3, angiopoietin-1, and sTie-2 may contribute to UI, though the mechanisms through which they work require further evaluation; however, these protein changes have been associated with endometriosis, raising the suggestion that subclinical/undiagnosed endometriosis may have contributed to UI in these subjects.

## 1. Introduction

Infertility is defined as the failure to establish a clinical pregnancy after 12 months of regular and unprotected sexual intercourse [1]. It affects approximately 8–12% of reproductive age couples worldwide, with male infertility being solely responsible for 20–30% of cases and contributing to up to 50% of cases [1]. Additionally, infertility may result in various psychological issues including depression, anxiety, and an overall decrease in quality of life. These factors have been observed to correlate with infertility independent of culture [2].

Male factor infertility (MFI) affects about 7% of the male population and, with over 2000 genes linked to spermatogenesis, genetic causes of infertility are complex though genetic anomalies in semen are increasingly being recognized [3]. Men with azoospermia have the highest risk of having genes (25%) affecting their fertility. Moreover, studies have shown that there are considerably higher copy number variant (CNV) loads in infertile versus normozoospermic men [3,4]. Among causes of MFI, varicoceles are the most common correctable cause, present in 45–81% of men with secondary infertility [5]. Other causes include endocrinopathies, genetics, cryptorchidism, exposure to gonadotoxins, obstruction, and defective ejaculation [5].

Unexplained infertility (UI) affects about 15–30% of couples and is defined as an inability to conceive for >12 months with no abnormality found on diagnostic workup [6]. In a study of patients with UI, the prevalence of endometriosis, diagnosed by exploratory laparoscopy, was 44%, and the prevalence of tubal factors and adhesions were 20% and 16%, respectively [6].

In couples affected by MFI and UI, in vitro fertilization (IVF) is often utilized to conceive.

Endothelial dysfunction is shown to be implicated as a factor contributing to infertility [7] and is linked to conditions affecting reproduction such as polycystic ovary syndrome (PCOS) and endometriosis [7,8].

**Figure 1:**
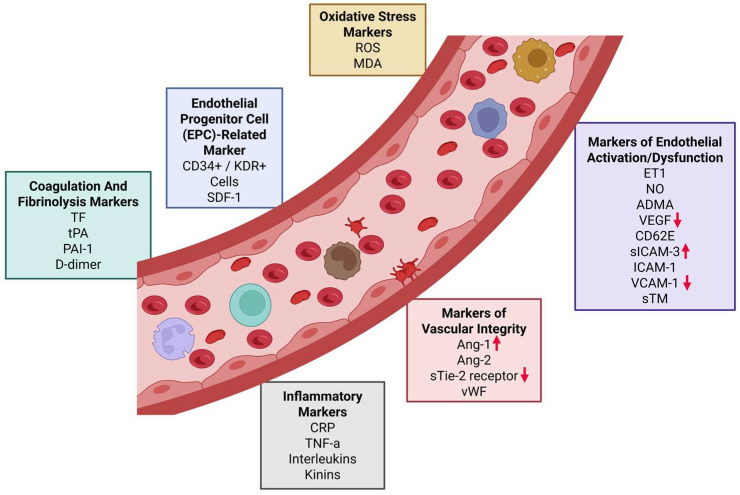
A schematic to categorize the proteins involved in endothelial function. In women with unexplained infertility, three markers of endothelial activation/dysfunction differed (VCAM-1, sICAM-3, and VEGF) and two markers of vascular integrity (angiopoetin-1 and sTie-2) compared to control women with male factor infertility as indicated by red arrows.

Endothelial factors may be grouped broadly by their functions, including coagulation and fibrinolysis markers, inflammatory markers, oxidative stress markers, markers of endothelial activation/dysfunction, markers of vascular integrity, and endothelial progenitor cell-related markers (Figure 1). In endothelial dysfunction, biomarkers of endothelial activation are often upregulated. Such markers include adhesion molecules like intercellular adhesion molecule 1 (ICAM-1), vascular cell adhesion molecule 1 (VCAM-1), and E-selectin [9]; growth factors like angiopoietin-1 (ANGPT1) and ANGPT2 [10]; and coagulation factors such as P-selectin, D-dimer, and fibrinogen [11,12]. Furthermore, imbalanced expression of endothelin-1, tissue plasminogen activator (tPA), plasminogen activator inhibitor-1 (PAI-1), and von Willebrand factor (vWF) are also characteristic of endothelial dysfunction.

Vitamin D deficiency has been shown to contribute to conditions such as endometriosis, PCOS, pregnancy complications, and infertility [13]. The role of vitamin D may be to modulate endothelial function as it is known to improve endothelial cell migration and proliferation through upregulated gene expression of matrix metalloproteinase 2 (MMP-2), an extracellular matrix-dissolving factor [13,14], suggesting that vitamin D levels should be measured when evaluating endothelial function and infertility.

This study aimed to compare the expression of endothelial protein factors in women undergoing IVF with either MFI or UI and to determine whether vitamin D_2_, D_3_, and 1,25 vitamin D_3_ differed between groups. We hypothesized that endothelial protein factors may provide evidence for the underlying mechanism in UI and differ from that in normal women with MFI.

## 2. Results

### Demographics, Metabolic Outcomes, and Hormone Levels

Age, body mass index (BMI), CRP, vitamin D_2_, vitamin D_3_, 1,25 vitamin D_3_, and AMH did not differ between MFI and UI (Table 1). There were no differences in reproductive outcomes for top-quality embryos (G3D3), the proportion of top-quality embryos [15], or in fertility rate (Table 1).

**Figure 2:**
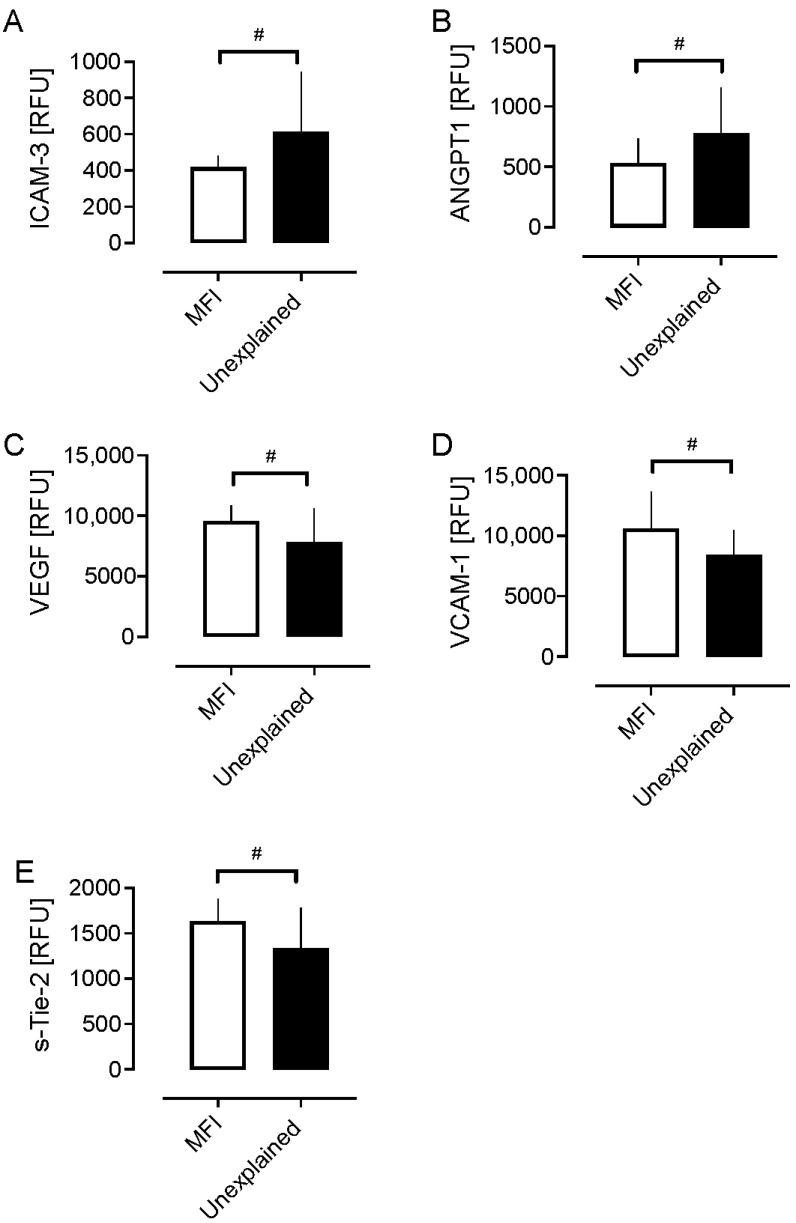
A comparison of endothelium marker levels in women with male factor infertility (MFI) compared to women with unexplained infertility; # *p* < 0.05. (**A**) ICAM-3, intercellular adhesion molecule 1; (**B**) ANGPT1, angiopoietin-1; (**C**) VEGF, vascular endothelial growth factor; (**D**) VCAM-1, vascular cell adhesion molecule 1; (**E**) s-Tie-2, angiopoetin-1 receptor. The data is presented as column bar graphs using GraphPad Prism v9.5.1 (San Diego, CA, USA) software. The data is presented as mean ± SD. The Student’s *t*-test was used to compare differences between groups.

In UI, markers of endothelial activation/dysfunction were investigated: VCAM-1 decreased (*p* < 0.05) and VEGF decreased (*p* < 0.05) in comparison with MFI subjects (Figure 1), while sICAM-3 increased (*p* < 0.05) as shown in Figure 2 and Table 2. In addition, in UI compared to MFI subjects, a marker of vascular integrity, angiopoietin-1, increased (*p* < 0.05) while sTie-2 decreased (*p* < 0.05) as shown in Figure 2. Endothelial markers of inflammation, coagulation, and endothelial progenitor cells were unchanged.

**Figure 3:**
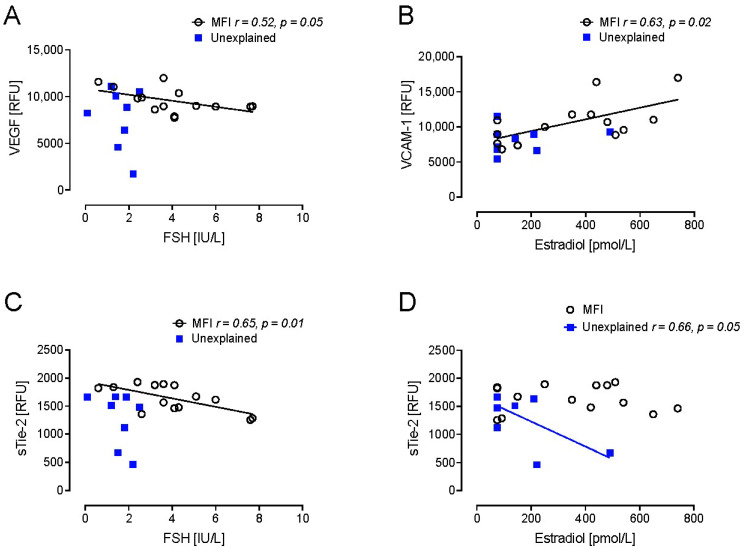
Correlations of endothelium marker levels in women with male factor infertility (MFI) compared to women with unexplained infertility, with follicle stimulating hormone, and estradiol. (**A**) VEGF, vascular endothelial growth factor correlated with FSH; (**B**) VCAM-1, vascular cell adhesion molecule 1 correlated with estradiol; (**C**) s-Tie-2, angiopoetin-1 receptor correlated with FSH; (**D**) s-Tie-2, angiopoetin-1 receptor correlated with estradiol. Simple linear regression was performed using GraphPad Prism v9.5.1 (San Diego, CA, USA) software. ‘Goodness of fit’ is indicated by the *r* value with significance taken at the level of *p* < 0.05.

Correlation with hormonal parameters at day 21 of LH, FSH, and estradiol showed that, in MFI, VEGF negatively correlated with FSH (*p* < 0.05), VCAM-1 positively correlated with estradiol (*p* < 0.05), and sTie-2 negatively correlated with FSH (*p* < 0.01), but these correlations were not found for UI (Figure 3). In UI, sTie-2 negatively correlated with estradiol while this was not seen for MFI (Figure 3).

Levels of vitamin D_2_, D_3_, and 1,25 vitamin D_3_ did not differ between the MFI and UI cohorts and did not correlate with hormonal parameters or the proteins associated with endothelial function.

## 3. Discussion

The novelty of this study was the finding of the combination of VCAM-1, VEGF, sICAM-3, angiopoietin-1, and sTie-2 protein changes, which have not been reported in UI at any time but rather individually in endometriosis, a condition that has been associated with UI. Of note, all of the subjects with UI had a diagnostic laparoscopy that was normal with no evidence of endometriosis. The markers of endothelial activation/dysfunction VCAM-1 and VEGF decreased, while sICAM-3 increased in UI; in addition, in UI, a marker of vascular integrity angiopoietin-1 increased while sTie-2 decreased. These findings are in accord with changes in endothelial dysfunction and vascular integrity alterations that have been reported in the pathophysiology of UI [8]. Studies report dysregulation of proteins such as vascular endothelial growth factor (VEGF), endothelial nitric oxide (eNOS), and adhesion molecules, which possibly reflect subclinical vascular impairment affecting endometrial receptivity, angiogenesis, or uterine tolerance [8,16,17].

VCAM-1, as a member of the integrin family, has a role in assisting with leukocyte adhesion and migration; however, underexpressed VCAM-1 in UI may indicate endothelial stress [18], reflecting impaired endothelial responsiveness, potentially affecting endometrial receptivity and implantation success. Lower VCAM-1 levels have been reported in women with endometriosis [19] that may be associated with UI [6]. Elevated VCAM-1 has also been associated with immunological disorders and, therefore, the decreased levels here may suggest that it is not an autoimmune process contributing to UI [20]. The results here are in accord with a report of lower VCAM-1 levels in the endometrium from women with UI [21].

VEGF is essential for angiogenesis and vascular permeability regulation, and its decrease in UI could suggest impaired endometrial angiogenesis, a critical factor for successful implantation [22]. Studies have reported the expression of VEGF in the endometrium throughout the menstrual cycle, with a significant increase in the mid-luteal phase, suggesting a role for endometrial VEGF around the time of embryo implantation [23], and the lower levels found at day 21 in the mid-luteal phase in this study may be indicative of an impaired VEGF response. In addition, lower serum VEGF levels have been reported in women with endometriosis [24].

sICAM-3, also called CD50, is constitutively expressed in all leukocytes, binds to lymphocyte function-associated antigen-1 (LFA-1) protein for its function, and mediates the removal of cells undergoing apoptosis [25]. Elevated sICAM-3 may be associated with increased immunological activity through leukocyte activation, and cell adhesion mechanisms specifically involved in human immunity [26]. Elevated sICAM-3 has been shown in endometriotic lesions [27] and the peritoneal fluid of women with endometriosis [28].

In addition to endothelial activation markers, alterations in vascular integrity markers were studied in the 25 participants. Ang-1 is an endothelial-specific growth factor that acts through tyrosine kinase receptor Tie2, which promotes vascular integrity and vascular development [29]. Ang-1 was significantly increased in UI, and it is noteworthy that Ang-1 mRNA is higher in the endometrium of women with endometriosis [30], suggesting increased vascular development. Increased levels of Ang-1 lead to increased vascular stability and maturation, reduced vascular permeability, and anti-inflammatory effects. Ang-1 primarily acts on the Tie2 receptor to promote these effects [29]. Tie2 is a decoy receptor that is expressed mainly on the surface of blood and lymphatic endothelial cells [29]. In this study, sTie-2 was decreased in UI sample studies. It may be expected that an increase in Ang-1 may reflect an increase in Tie2, but in a study showing that Ang-1 mRNA is higher in the endometrium of women with endometriosis [30], Tie2 levels were unchanged compared to normal. It has been reported that Tie2 may be regulated by VEGF; therefore, the lower VEGF levels seen here may have been reflected in lower Tie2 [31].

In the MFI cohort, VEGF negatively correlated with FSH, VCAM-1 positively correlated with estradiol, and sTie2 negatively correlated with FSH, but these correlations were lost in UI. Conversely, sTie2 correlated with estradiol in UI but not in MFI, suggesting that there are likely changes in the hypothalamo-gonadal axis in UI in comparison to the “normal” comparator MFI [32]. Hormonal changes may also be associated with inflammatory responses or endothelial activation as reflected by the positive correlation between VCAM-1 and estradiol in MFI [8,33].

The reduction in VCAM-1 and VEGF, and the increase in sICAM-3 and Ang-1, have all been associated with endometriosis and, while no subject with UI had overt endometriosis, it is interesting to postulate that these women may have subclinical endometriosis, which would be in accord with the high prevalence of endometriosis reported in UI [6].

FSH levels have been reported to be higher in endometriosis through an association with decreased ovarian reserve [34], and in this study it is of interest that there was an inverse relationship between FSH levels and both VEGF and Tie-2 levels that were shown to be lower in UI, though the mechanism behind this and whether these proteins are associated with ovarian reserve require further evaluation. Estrogen levels can be higher in endometriosis [35] and were positively correlated with VCAM-1 and negatively with Tie-2 levels, but more research needs to be undertaken to understand the significance of these findings that suggest these proteins are involved in the reproductive axis.

Vitamin D deficiency has been shown to contribute to conditions such as endometriosis and infertility [13]. Given the observed endothelial protein changes, therapeutic strategies targeting vascular function may hold promise for improving fertility outcomes in UI. Recent studies have shown that vitamin D promotes endothelial cell adhesion and proliferation by upregulating matrix metalloproteinase-2 (MMP-2), an extracellular matrix-dissolving factor [13,14]. However, in this study, there were no differences in vitamin D levels between either the UI or MFI groups, suggesting that the differences reported here between UI and MFI subjects were independent of vitamin D.

Metformin is a potential therapeutic regimen that addresses endothelial dysfunction by improving insulin resistance and limiting inflammatory and oxidation-responsive pathways [36]. Metformin treatment of endometriosis has been used, which decreases the expression levels of critical inflammatory and angiogenesis-related genes including VEGF, MMP2, and MMP9, while stimulating tissue inhibitors of metalloproteinase, endometrial stromal cells, and progesterone receptor expression [37]. Other potential therapeutic approaches could include a combination of first- and second-generation anti-angiogenic agents targeting the angiopoietin–Tie-2 pathway, aimed at restoring VEGF levels [38]. Incorporation of recommended lifestyle modifications of exercise and dietary changes that may improve endothelial function may also be explored as adjunct strategies for managing UI [39].

Given that there is not a diagnostic blood test for endometriosis, there has been interest in identifying biomarkers for the condition [40] and, while there have been over 400 articles with control groups, only four (TNF-a, MMP-9, TIMP-1, and miR-451) are reported to be detected in at least three tissues [40], suggesting that further research on endothelial proteins needs to be explored in endometriosis.

It is well recognized that infertility may be caused by a combination of both male and female issues [1]. In this study, subjects were investigated to exclude any other factors such that only a significant male factor issue was present in the MFI group; then using this group as the control subjects, investigation of the UI group, including diagnostic laparoscopy, gave no indication as to why they could not conceive. It cannot be absolutely excluded that those with MFI did have an additional female factor. The strengths of this study lie in the well-matched MFI and UI group sample for age and BMI. In addition, all these women were undergoing an IVF program for fertility, none had been on any hormonal contraception, and they had all stopped any alcohol consumption and all were non-smokers, so these confounders could be excluded from the analysis. It is well recognized that endothelial function differs through the menstrual cycle, and this confounder was mitigated by all women being at day 21 of their menstrual cycle [41]. Limitations include the small sample size of the study and a type 2 statistical error that may be of statistical significance with a larger cohort. Only a Caucasian population was used, and the study needs to be repeated with a more ethnically diverse population. The only difference in the investigation between the MFI and the UI subjects was that the UI subjects had a diagnostic laparoscopy while the MFI subjects did not. In addition, while subclinical/undiagnosed endometriosis as an underlying factor responsible for unexplained infertility is not a novel observation per se, the unexpected combination of VCAM-1, VEGF, sICAM-3, angiopoietin-1, and sTie-2, which are all proteins that have been associated with endometriosis, is novel and compelling; however, a causal association between these proteins, endometriosis, and UI requires more study, and the exclusion of other mechanisms that are out of the scope of this study.

## 4. Materials and Methods

The study was designed as a case–control study. Twenty-nine women were recruited sequentially from the In Vitro Fertilization (IVF) Unit, Hull, UK, in 2015. Ethical approval was obtained from The Yorkshire and The Humber NRES ethical committee, UK (approval number 02/03/043); approved February 2003. Consent forms for participation were distributed to all participants and signed by each participant. Exclusion criteria were documented immunological or inflammatory disease, infection (acute or chronic), liver/renal insufficiency, diabetes, body mass index (BMI) > 30 kg/m^2^, age outside the 20–45-year range, and anyone not undergoing IVF treatment. From review of their medical histories, none of the women had been taking any over-the-counter or prescription medication, all were nonsmokers, and all had abstained from alcohol for >6 months. Investigation of the UI subjects included an exploratory laparoscopy.

All subjects commenced their IVF treatment during the subsequent cycle following a short antagonist IVF protocol. rFSH stimulation was initiated on day 2 of the menstrual cycle with either Merional (Pharmasure, Watford, UK) or Gonal-F (Merck Serono, Middlesex, UK). The dose of stimulation was based on AMH, antral follicle count, age, and the ovarian response of previous treatment. A dose of 0.25mg/day Cetrotide (GnRH antagonist; Merck Serono, Middlesex, UK) was commenced on day 6 of stimulation (for premature LH surge prevention). When ≥2 leading follicles were ≥18mm, triggering of final maturation was induced with either 0.5mg Buserelin (Sanofi-Aventis, Frankfurt, Germany) or 5000–10,000 IU human chorionic gonadotrophin (hCG, Pregnyl (Merck Sharp and Dohme). Oocyte retrieval was undertaken 36 h later transvaginally, and luteal support was effected using 600 mg of nightly progesterone (Uterogestan, Besins Iscovesco Laboratories, Paris, France) beginning on the oocyte retrieval day. Embryo transfers were undertaken either on day 3 or preferably on day 5 (blastocyst) to provide the optimal chance for successful implantation. Embryos were categorized using standardized criteria [42] at the cleavage and blastocyst stages.

### 4.1. Sample Collection

At day 21 of the menstrual cycle, before IVF treatment was commenced, and when no hormonal therapy had been administered, mock embryo transfer was performed (as is standard clinical practice) together with ovarian/endometrial ultrasound. Fasting blood was collected on that visit and centrifuged (3500× *g* for 15 min at 4 °C) with subsequent storage (−80 °C). A Synchron LX20 analyzer (Beckman-Coulter, Brea, CA, USA) was used to measure fasting blood glucose (FBG). A competitive chemiluminescent immunoassay (DPC Immulite 2000 analyzer, Euro/DPC, Llanberis, UK) was used to measure serum insulin. Calculation of the homeostatic model assessment for insulin resistance (HOMA-IR) was performed using the following formula: ((Insulin × glucose)/22.5) [43]. A chemiluminescent microparticle immunoassay (Abbott Diagnostics, Maidenhead, UK) was used to determine serum progesterone, estradiol, luteinizing hormone (LH), and follicle stimulating hormone (FSH). Enzymatic determination of total cholesterol (TC), triglycerides (TG), and C-reactive protein (CRP) was undertaken (Synchon LX20 analyzer, Beckman-Coulter). The following formula was used to determine total serum lipid (TSL): ((2.27 × TC) + TG + 62.3 mg/dL) [44]. An immunoenzymatic assay (Beckman-Coulter) was utilized to measure anti-Müllerian hormone (AMH). Ion-exchange chromatography was utilized to determine glycosylated hemoglobin A1c (HbA1c). The following formula was used to calculate estimated glomerular filtration rate (eGFR): ((175 × (SCr, mg/dL) − 1.154 × (age, years) − 0.203 × 0.742)) [45]. Liquid chromatography tandem mass spectrometry (LC/MS/MS; Acquity UPLC-Quattro Premier XE-MS, Waters, Manchester, UK) was used to measure androgens. Sex hormone-binding globulin (SHBG) was determined by an immunometric assay with fluorescence detection (DPC Immulite 2000 analyzer; upper limit 2.0 nmol/L). The following formula was employed to calculate the free androgen index (FAI): ((testosterone/SHBG) × 100). Thyroid hormone levels were determined using an Abbott Architect i4000 immunoassay analyzer (Abbott Diagnostics Division, Maidenhead, Berkshire, UK).

Plasma protein levels, targeting proteins associated with endothelial function, were determined using the Slow Off-rate Modified Aptamer (SOMA)-scan platform [46] as has previously been described [47,48]. Using EDTA plasma samples, the following steps were undertaken: (1) analyte/primer beads bound to SOMAmers (synthetic SOMAmers with fluorescent labeling were bound to biotin through a photocleavable linker) were equilibrated; (2) a streptavidin-substituted matrix was employed and immobilization of the analyte/SOMAmer complexes was achieved; (3) cleavage was effected with UV light, releasing the analyte/SOMAmer complexes into the solution; (4) analyte/SOMAmer complex immobilization was effected through analyte-borne biotinylation upon a streptavidin matrix; (5) complexes were eluted releasing SOMAmers (that serve as surrogates for analyte quantification); (6) SOMAmer-complementary oligonucleotide hybridization enabled quantification. Calibration standards were employed as previously described [49]. Standardization with normalization of raw intensities, hybridization, and median and calibration signal analysis was performed [46,49].

We used version 3.1 of the SOMAscan Assay, specifically targeting those endothelial biomarker proteins in the SOMAscan panel (SomaLogic, Boulder, CO, USA). Markers of endothelial activation/dysfunction included vascular endothelial growth factor (VEGF), E-selectin, intercellular adhesion molecule (ICAM) 1,2,3,5, vascular cell adhesion molecule 1 (VCAM-1), P-selectin, and cadherin-5. Markers of vascular integrity included angiopoietin-1 (Ang-1) and angiopoietin-2 (Ang-2), Tie-2 receptor, and von Willebrand factor (vWF). Inflammatory markers included C-reactive protein (CRP); tumor necrosis factor-alpha (TNF-α); and pro-inflammatory cytokines, interleukins IL-1 and IL-6. Markers of coagulation and fibrinolysis included tissue factor (TF), tissue plasminogen activator (tPA), plasminogen activator inhibitor-1 (PAI-1), and D-dimer. Markers of vascular integrity included angiopoietin-1 (Ang-1) and angiopoietin-2 (Ang-2), Tie-2 receptor, and von Willebrand factor (vWF) (Figure 1).

### 4.2. Statistical Analysis

No published studies are available that could allow for performing a power calculation; therefore, we undertook an exploratory pilot study. Descriptive data (mean ± standard deviation (SD)) are presented for the continuous data. Endothelial protein level as well as metabolic/hormone levels were assessed for normality using the Kolmogorov–Smirnov statistical test; because for all proteins the *p* value of the K-S test was greater than 0.05, indicating likely normal distribution of the data, the Student’s *t*-test was used to compare differences between groups. Data is presented as mean (SD). Significance of the Student’s *t*-test was taken at the *p* < 0.05 level. Correlations between endothelial protein levels and metabolic parameters were investigated with Pearson’s correlations. A *p* value of 0.05 or less was taken to be statistically significant. For statistical analysis, Graphpad Prism v9.5.1 (San Diego, CA, USA) was utilized.

## 5. Conclusions

In conclusion, vitamin D and its metabolites show no relationship to UI, but endothelial activation/dysfunction and vascular integrity changes in VCAM-1, VEGF, sICAM-3, angiopoietin-1, and sTie-2 may contribute to UI, though the mechanisms require further evaluation; however, these protein changes have been associated with endometriosis, raising the suggestion that subclinical/undiagnosed endometriosis may have contributed to UI in these subjects.

## Figures and Tables

**Table 1:** Demographic and biochemical data in male factor (*n* = 14) and unexplained (*n* = 11) infertility. Data is presented as mean (SD). Student’s *t*-test was used to determine any differences between groups.

	Male Factor	Unexplained	*p* Value
Age (years)	32.6 (4.0)	33.8 (5.3)	0.51
BMI (kg/m^2^)	25.7 (2.6)	25.3 (4.9)	0.84
CRP (mg/L)	1.9 (1.3)	2.4 (2.0)	0.51
Vitamin D_2_ (ng/mL)	0.9 (0.4)	(0.7 (0.5)	0.62
Vitamin D_3_ (ng/mL)	20.9 (11.6)	23 (12.5)	0.68
1,25 vitamin D_3_ (ng/mL)	0.05 (0.02)	0.04 (0.01)	0.21
AMH (ng/mL)	22.4 (15.3)	24.5 (12.5)	0.72
Positive pregnancy test	0.3 (0.5)	0.3 (0.5)	0.95
Number of eggs retrieved	9.0 (7.5)	8.4 (3.2)	0.78
Number of embryos created	3.7 (3.0)	5.2 (2.4)	0.18
G3D3	3.4 (2.2)	2.7 (2.6)	0.49
Fertility rate	0.6 (0.2)	0.6 (0.4)	0.89
Top-quality embryo (proportion)	0.3 (0.2)	0.4 (0.4)	0.36
Live birth rate	0.0 (0.0)	0.0 (0.0)	1.0

BMI = body mass index, CRP = C-reactive protein, AMH = anti-Müllerian hormone, G3D3 = top-quality embryos day 3 as per alpha consensus [15].

**Table 2:** Endothelial cell protein marker levels in women with male factor (*n* = 14) and unexplained (*n* = 11) infertility. The data is expressed in relative fluorescent units (RFUs). The Kolmogrov–Smirnov (K-S) test was used to determine normality; because in all cases the *p* value of the K-S test was greater than 0.05, indicating likely normal distribution of the data, the Student’s *t*-test was used to compare differences between groups. The data is presented as mean (SD). Significance of the Student’s *t*-test was taken at the *p* < 0.05 level. The *p* values and D values of the K-S test used to determine normal distribution of the data are shown in the two columns on the right. * *p* < 0.05.

	Male Factor	Unexplained	*p* Value	Kolmogorov–Smirnov Test *p* Value D Value
sE-Selectin	23,045 (9127)	19,076 (6989)	0.25	0.12 0.48
sICAM-1	3239 (1231)	2949 (1058)	0.54	0.70 0.29
VCAM-1	10,615 (3019)	8415 (2022)	0.049 *	0.15 0.46
Cadherin-5	12,100 (2517)	10,402 (1696)	0.07	0.12 0.48
P-Selectin	13,288 (3141)	16,620 (11,235)	0.30	0.72 0.28
vWF	7008 (6708)	9222 (4813)	0.37	0.09 0.50
sICAM-3	419 (61)	614 (329)	0.04 *	0.22 0.42
sICAM-5	592 (117)	531 (163)	0.29	0.67 0.29
sICAM-2	1362 (125)	1923 (1099)	0.07	0.16 0.45
VEGF	9545 (1298)	7808 (2791)	0.050 *	0.22 0.42
Angiopoietin-1	531 (202)	775 (377)	0.049 *	0.33 0.38
Angiopoietin-2	93 (27)	93 (39)	0.97	0.44 0.35
sTie-2	1638 (240)	1335 (443)	0.03 *	0.21 0.43
TNF-a	343 (36)	350 (139)	0.87	0.37 0.37
IL-1a	688 (369)	622 (160)	0.59	0.95 0.21
IL-6	261 (70)	277 (65)	0.56	0.31 0.39
TF	940 (266)	1008 (597)	0.71	0.75 0.27
tPA	298 (165)	276 (123)	0.72	0.64 0.30
PAI-1	225 (160)	334 (227)	0.17	0.67 0.29
D-dimer	13,615 (2828)	13,790 (2971)	0.88	0.95 0.21
SDF-1	4451 (666)	4525 (1162)	0.84	0.59 0.31

## Data Availability

The data that support the findings of this study are available from the corresponding author, [A.E.B.], upon reasonable request.

## Abbreviations

CRP	C-reactive protein
TNF-a	tumor necrosis factor alpha
TF	tissue factor
tPA	tissue plasminogen activator
PAI-1	plasminogen activator inhibitor-1
ROS	reactive oxygen species
MDA	malondialdehyde
KDR	vascular endothelial growth factor receptor 2
SDF-1	stromal cell-derived factor 1
ANG-1	angiopoietin-1
ANG-2	angiopoietin-2
Tie-2	angiopoietin-1 receptor
vWF	von Willebrand factor
ET1	endothelin 1
NO	nitric oxide
ADMA	asymmetric dimethylarginine
VEGF	vascular endothelial growth factor
CD62E	E-selectin
ICAM-1	intercellular adhesion molecule 1
VCAM-1	vascular cell adhesion molecule 1
sTM	soluble thrombomodulin
